# Neural-network analysis of socio-medical data to identify predictors of undiagnosed hepatitis C virus infections in Germany (DETECT)

**DOI:** 10.1186/s12967-019-1832-4

**Published:** 2019-03-19

**Authors:** Markus Reiser, Bianka Wiebner, Jürgen Hirsch, the German Liver Foundation

**Affiliations:** 1 Dept. of Internal Medicine & Gastroenterlogy, Klinikum Vest GmbH – Paracelsus-Klinik Marl, Lipper Weg 11, 45772 Marl, Germany; 2 Deutsche Leberstiftung (German Liver Foundation), Carl-Neuberg-Str. 1, 30625 Hannover, Germany; 3 Hirsch Consulting, Steinbacher Str. 10, 65760 Eschborn, Germany

**Keywords:** Chronic hepatitis C, Screening, Artificial neural network, Data mining, Artificial intelligence

## Abstract

**Background:**

Chronic hepatitis C virus (HCV)-infection is a slowly debilitating and potentially fatal disease with a high estimated number of undiagnosed cases. Given the major advances in the treatment, detection of unreported infections is a consequential step for eliminating hepatitis C on a population basis. The prevalence of chronic hepatitis C is, however, low in most countries making mass screening neither cost effective nor practicable.

**Methods:**

We used a Kohonen artificial neural network (ANN) to analyze socio-medical data of 1.8 million insurants for predictors of undiagnosed HCV infections. The data had to be anonymized due to ethical requirements. The network was trained with variables obtained from a subgroup of 2544 patients with confirmed hepatitis C-virus (HCV) infections excluding variables directly linked to the diagnosis of HCV. All analyses were performed using the data mining solution “RayQ”. Training results were visualized three-dimensionally and the distributions and characteristics of the clusters were explored within the map.

**Results:**

All 2544 patients with confirmed chronic HCV diagnoses were localized in a clearly defined cluster within the Kohonen self-organizing map. An additional 2217 patients who had not been diagnosed with hepatitis C co-localized to the same cluster, indicating socio-medical similarities and a potentially elevated risk of infection. Several factors including, age, diagnosis codes and drug prescriptions acted only in conjunction as predictors of an elevated HCV risk.

**Conclusions:**

This ANN approach may allow for a more efficient risk adapted HCV-screening. However, further validation of the prediction model is required.

## Background

Chronic hepatitis C virus (HCV)-infection is a slowly progressive disease which may lead to end-stage liver disease including liver cirrhosis and hepatocellular carcinoma [1]. Due to the often silent course of the acute as well as chronic infection the diagnosis may be missed [2]. Globally, an estimated 70 million people are chronically infected with the hepatitis C virus of whom only 20% are believed to be diagnosed [3]. Among those, treatment has been initiated in only a small fraction; in 2015, 1.1 million or 7.4% of patients diagnosed with chronic hepatitis C started treatment [4].

The cumulative number of patients who have received treatment over the years reached 5.4 million in 2015. Most of these patients received older, interferon-based therapies characterized by substantial side effects and lower sustained elimination rates approximating 50% [5]. With the recent introduction of several direct-acting antivirals (DAAs) treatment of chronic HCV infection has become highly effective with cure rates approaching 100% [6]. Moreover, the excellent safety and low side effect profiles of DAAs make treatment even of advanced disease states feasible.

In May 2016, the World Health Assembly endorsed the Global Health Sector Strategy (GHSS) on viral hepatitis 2016–2021. The GHSS calls for the elimination of viral hepatitis as a public health threat by 2030 [7]. To achieve this goal, detection and treatment of hitherto undiagnosed HCV infections is vital. Despite its major health care implications, the overall low prevalence of chronic HCV-infection between 0.5 and 2.3% makes mass screening not practicable since a high number of healthy individuals would be tested. This also applies to Germany with an estimated HCV antibody and viremic prevalence of 0.3–0.9% and 0.2–0.64% respectively [8, 9]. This study, initiated by the German Liver Foundation (Deutsche Leberstiftung), therefore sought to describe a large group of patients with proven hepatitis C and to identify predictors for undiagnosed HCV infections by analyzing socio-medical data provided by two German health care insurance companies. Anonymized health care data of 1.8 million individuals collected between 2009 and 2014 were subjected to an in depth ANN analysis in order to define a cluster of patients showing socio-medical similarities with a subgroup of confirmed HCV diagnoses, indicating a potentially elevated risk of undiagnosed infections. This novel approach may allow for a more efficient risk-adapted HCV-screening.

## Methods

The study was approved by the institutional research ethics committee and performed in accordance with the ethical standards as laid down in the 1964 declaration of Helsinki and its later amendments. The ethics committee required all data to be anonymized and did not allow for the retracing of patients who might be at risk for HCV-infection in order to offer testing. This was justified by the *right not to know*. Therefore, the study protocol proposed to distribute all information gained through public relations and the support of the German Liver Foundation in order to sensitize patients and health care providers about diagnostic and therapeutic measures. Moreover, after obtaining informed consent the prediction model may be applied by physicians and health insurance companies in the future.

### Data acquisition and analysis

Insurance and treatment data of 1.82 million persons covering the observation period between 2009 and 2014 were provided by two German private health insurance companies: DEBEKA (Debeka Krankenversicherungsverein a. G.) and HUK Coburg (HUK-COBURG-Krankenversicherung AG).

The following variables were available for analysis: age, sex, ZIP code of residence, prescription medications given as Pharmazentralnummer (PZN, which is a nationwide standardized identification number for medicines) and ATC code (anatomical therapeutic chemical classification system), outpatient and inpatient diagnoses (coded according to ICD 10), length of hospital stay, number of disability days and requirement of ambulatory care.

All analyses were performed using the data mining solution “RayQ” which supports the full process chain from accessing data pools to producing final reports in one software (http://www.rayq.info). RayQ provides neural network performance software and has been used for complex analyses in the public health sector for many years.

Patients with a confirmed diagnosis of chronic hepatitis C-virus infection were extracted from the data pool and described by descriptive statistics. Learning variables for training the ANN were selected from this group on the basis of potential HCV-associated indicators such as clues of extrahepatic disease manifestations [10]. These variables were derived from published studies and personal experience of extrahepatic HCV-related disorders and their treatment such as diabetes, depression, fatigue, arthralgia and thyroid diseases. Of note, no variables directly linked to the diagnosis of HCV (e.g. diagnosis of hepatitis or cirrhosis, drug treatment with interferon or direct antiviral agents, hepatocellular carcinoma) were chosen to train the ANN. The learning variables are listed in Table 1. The data were then converted to a uniform and linear measure of value before training the ANN. A clustering analysis was then performed on the entire cohort of 1.8 million insurants using Kohonen self-organizing maps (SOM) of varying sizes and training epochs to check if the clustering was random or solid. Training results were visualized three-dimensionally and the distributions and characteristics of the clusters were explored in the map. The ANN was tested with 8 × 8, 16 × 16, 32 × 32 and 64 × 64 neurons. Smaller sizes showed the cluster but did not represent the colors exactly. On the other hand, larger nets showed dead neurons, therefore a size of 32 × 32 was the best fit. The central questions were (1) do the known HCV-diagnoses cluster in a defined region within the SOM and (2) how many patients show high dimensional similarities with this cluster of known infections throughout all variables.

**Table 1:** Learning variables for training the ANN were selected from this group on the basis of potential HCV-specific indicators such as clues of extrahepatic disease manifestations [10]

Variables
Age (learn only, no best match)
Gender (learn only, no best match)
Relative frequency of HCV-infections within 2-digit ZIP codes
Frequency of drug prescriptions by ATC for
Levothyroxine (n = 5450)
Ibuprofen (n = 3534)
Metamizol (n = 2790)
Diclofenac (n = 2324)
Diclofenac topical (n = 2112)
Zopiclon (n = 1854)
Prednisolone (n = 1798)
Metformin (n = 1517)
Frequency of four common drug prescriptions by PZN for
Cholecalciferol (n = 473)
Decristol 20.000 iE (n = 473)
Lyrica 75 mg (n = 473)
Pregabalin (n = 369)
Frequency of out-patient diagnoses
E04.9 nontoxic goitre, unspecified (n = 1123)
M81.9 osteoporosis, unspecified (n = 1122)
F32.9 major depressive disorder, single episode (n = 1073)
L30.9 dermatitis, unspecified (n = 1039)
M54.5 low back pain (n = 1016)
E14 diabetes mellitus, unspecified (n = 948)
R53 malaise and fatigue (n = 743)
E1190 diabetes mellitus, not primary insulin dependent (n = 728)
One HCV uncorrelated but in the HCV group statistically heaped diagnosis
H52.2 astigmatism
Average length of hospital stay

Of note, no variables directly linked to the diagnosis of HCV (e.g. diagnosis of hepatitis C, drug treatment with interferon or direct antiviral agents) were chosen

Hepatitis C virus-infections newly diagnosed in 2014 (the end of the observation period) were separately examined to answer whether these infections could have been predicted earlier by the proposed ANN algorithm.

## Results

The cohort included 1,819,646 persons living in 86 different German ZIP code regions (defined by the first two of five ZIP code digits) with treatment data between 2009 and 2014. A total of 2544 patients (0.14%) were diagnosed with hepatitis C virus infection during the study period; 225 HCV infections were newly diagnosed in 2014. The demographic characteristics and treatment variables of the persons studied are shown in Table 2.

**Table 2:** Cohort characteristics

Characteristic	Known HCV-infection	All persons
Persons (no.)	2544	1,819,646
Male sex (%)	51	51
Average age at end of 2015 (year)	60.4	46.6
Prescriptions (no.)	209,727	175,369,031
Inpatient treatments (no.)	2343	
Length of hospital stay (days)	21.9	
No. of outpatient diagnoses	219,418	
Ambulatory care (%)	132 (5.2%)	

### Patients with known hepatitis C-virus infections

The average age of the patients with a known chronic HCV-infection was 60.4 years, 13.8 years older than the total group of insurants. In-hospital treatments were observed in 2343 of the 2544 (92%) HCV-infected patients with a total of 32,810 treatment days during the observation period. The average length of in-hospital stay was 21.9 days. The 20 most frequent main diagnoses are shown in Table 3. In the outpatient setting a total of 219,418 diagnoses were made. The 20 most frequent outpatient diagnoses are shown in Table 4. 132 patients (5.2%) with a known HCV-infection received ambulatory care. There were 209,727 prescriptions during the study period, the most frequent ATC codes are shown in Table 5.

**Table 3:** The 20 most frequent in-hospital diagnoses in the subgroup of HCV-infected patients

ICD-10	Description	Frequency
Z92.9	Personal history of medical treatment, unspecified	98
K74.6	Other and unspecified cirrhosis of liver	68
B18.2	Chronic viral hepatitis C	66
Z38.0	Singleton, born in hospital	54
C22.0	Liver cell carcinoma	48
G47.3	Sleep apnoea	47
M16.1	Other primary coxarthrosis	41
I48.10	Persistent atrial fibrillation	39
I20.0	Unstable angina	39
M17.1	Other primary gonarthrosis	36
K40.9	Unilateral or unspecified inguinal hernia, without obstruction or gangrene	36
K70.3	Alcoholic cirrhosis of liver	27
I20.8	Other forms of angina pectoris	27
K57.32	Diverticular disease of large intestine without perforation or abscess	26
M51.1	Lumbar and other intervertebral disc disorders with radiculopathy	25
R55	Syncope and collapse	24
I10	Essential (primary) hypertension	23
N39.0	Urinary tract infection, site not specified	22
H25.1	Senile nuclear cataract	22
N20.1	Calculus of ureter	21

*ICD-10* International Statistical Classification of Diseases and Related Health Problems 10th revision

**Table 4:** The 20 most frequent out-patient diagnoses in the subgroup of HCV-infected patients

ICD-10	Description	Frequency
B18.2	Chronic viral hepatitis C	6590
I10	Essential (primary) hypertension	4694
H52.2	Astigmatism	4184
H52.4	Presbyopia	3519
H52.1	Myopia	2043
K75.9	Inflammatory liver disease, unspecified	1699
H61.2	Impacted cerumen	1544
Z00.0	General medical examination	1473
H26.9	Cataract, unspecified	1435
Z01.7	Laboratory examination	1382
M54.2	Cervicalgia	1332
E78.0	Pure hypercholesterolaemia	1318
M17.9	Gonarthrosis, unspecified	1288
J06.9	Acute upper respiratory infection, unspecified	1280
N89.8	Other specified noninflammatory disorders of vagina	1256
J40	Bronchitis, not specified as acute or chronic	1270
B99	Other and unspecified infectious diseases	1184
E78.5	Hyperlipidaemia, unspecified	1125
E04.9	Nontoxic goitre, unspecified	1123
M81.9	Osteoporosis, unspecified	1122

**Table 5:** The 20 most frequent prescriptions within the study period (ATC code)

ATC code	Generic Drug	Frequency
H03AA01	Levothyroxine	5450
A02BC02	Pantoprazole	5237
C07AB02	Metoprolol	3697
M01AE01	Ibuprofen	3534
B01AC06	Acetylsalicylic acid	3382
C07AB07	Bisoprolol	3124
N02BB02	Metamizole	2790
C09AA05	Ramipril	2696
C08CA01	Amlodipine	2669
A05BA03	Silymarin	2591
M01AB05	Diclofenac	2324
M02AA15	Diclofenac	2112
C03CA04	Torasemid	2048
N05CF01	Zopiclone	1854
C10AA01	Simvastatin	1844
H02AB06	Prednisolone	1798
C09CA06	Candesartan	1543
A10BA02	Metformin	1517
J01MA02	Ciprofloxacin	1511
A02BC01	Omeprazole	1495

### Prediction model to identify patients with chronic hepatitis C

**Fig. 1:**
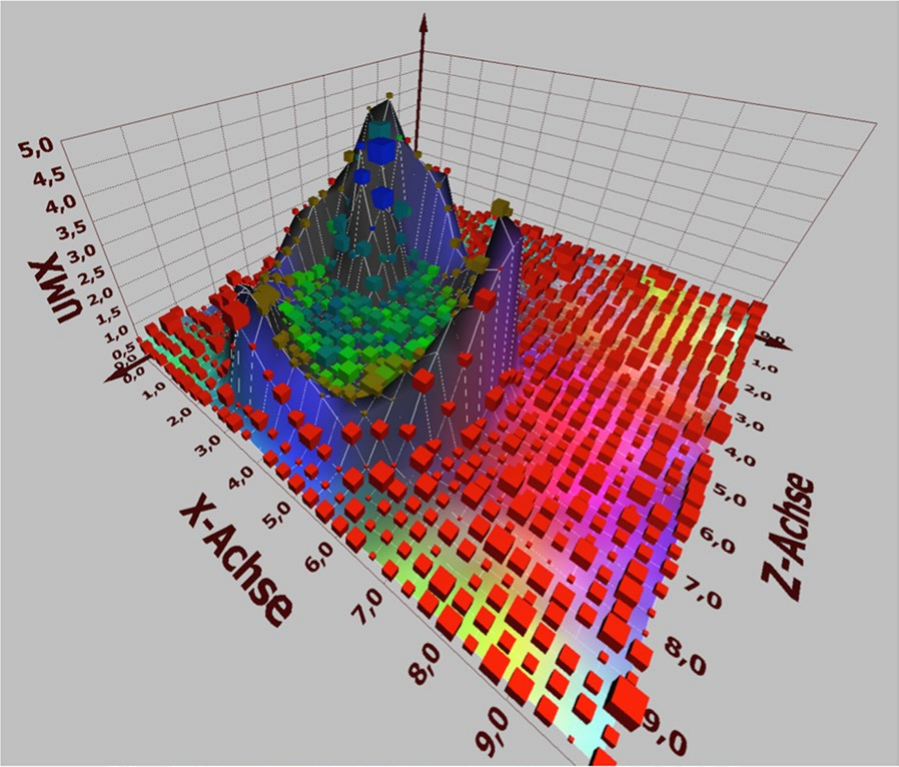
The Kohonen self organizing map (SOM) generated through 20 learning epochs. A clearly defined cluster is formed including all patients with a confirmed HCV-diagnosis: green cubes: confirmed and suspected HCV-infections diagnosed before 2014; blue cubes: confirmed HCV-infections diagnosed in 2014; blue–green cubes: confirmed and suspected HCV-infections diagnosed during the entire study period; brown cubes suspected HCV-infections. Red cubes represent patients without a confirmed or suspected HCV-infection. Of note, no confirmed HCV-infections are found outside the cluster

**Fig. 2:**
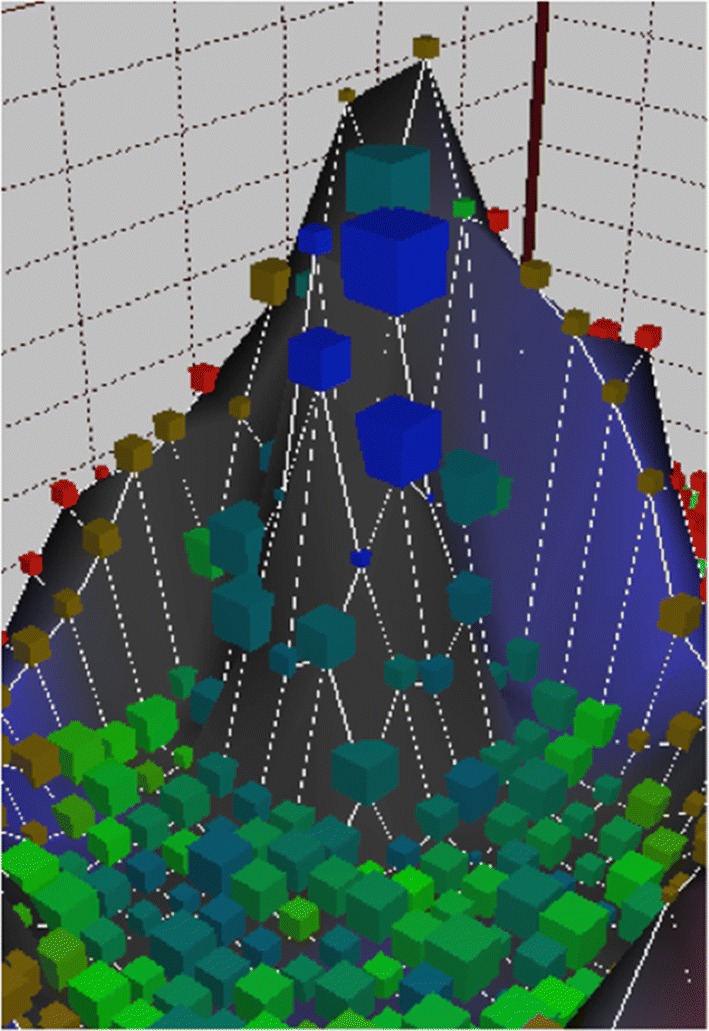
Detail from Fig. 1 showing the cluster of confirmed and suspected HCV-infections (blue, green and blue-green cubes)

**Fig. 3:**
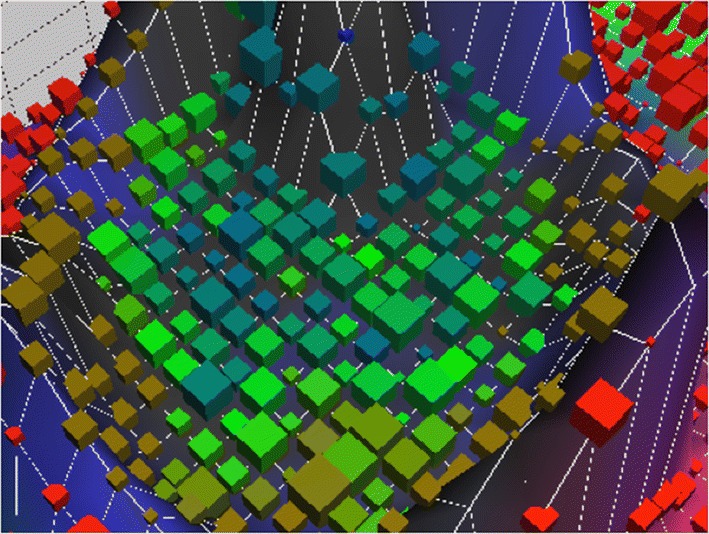
Detail from Fig. 1 showing the cluster of confirmed and suspected (green and blue-green cubes) and suspected HCV-infections (brown cubes)

The data of all patients were imported into the ANN for 20 learning epochs, thereby generating a Kohonen SOM disclosing non linear correlations between high dimensional data within a three-dimensional surface (Fig. 1). Data sets were depicted within the map as cubes of different sizes according to the quantity of contained data. The distance between objects represents the degree of similarities based on the variables used to train the ANN. A clearly defined cluster formed in the SOM and could further be subdivided into different patient subclusters (Figs. 1, 2 and 3): green cubes: confirmed and suspected HCV-infections diagnosed before 2014; blue cubes: confirmed HCV-infections diagnosed in 2014; blue–green cubes: confirmed and suspected HCV-infections diagnosed before or in 2014 (during the entire study period); brown cubes suspected HCV-infections. Red cubes represent patients without a confirmed or suspected HCV-infection. Of note, no confirmed HCV-infections are found outside the cluster. These results were reproducible also when varying the parameters of the ANN such as the number of nodes or learning epochs.

136 of the 225 HCV-infections (60%) diagnosed in 2014 were allocated to a distinct location (blue cubes) with the vector pointing towards the cluster center. 2166 patients clustered within the green and blue–green cubes, 2459 patients were found within the brown cubes. Therefore, a total of 4761 confirmed or suspected HCV-infections were identified through the SOM (136 + 2166 + 2459). Since this figure includes the 2544 known HCV-infections, 2217 patients have not been diagnosed with HCV but show strong similarities with the group of confirmed infections based on the socio-medical data analyzed.

### Kohonen recall analysis

**Fig. 4:**
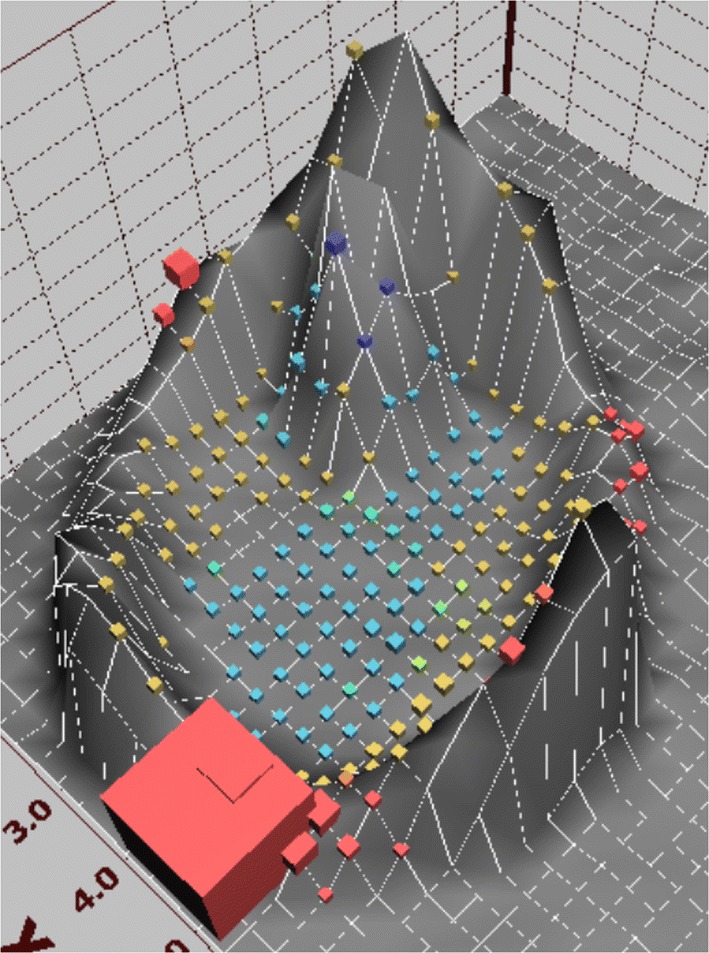
Kohonen recall analysis to verify the prediction model generated by the SOM. After completion of the learning cycles the map was frozen and the original data were imported against the trained map. Again, all patients with a confirmed or suspected diagnosis of HCV-infection are relocated within the defined cluster

In order to verify the prediction model generated by the SOM a Kohonen recall was performed: After completion of the learning cycles the map was frozen and the original data were imported against the trained map. Again, all patients with a confirmed or suspected diagnosis of HCV-infection were relocated within the defined cluster (Fig. 4).

### Comparison of groups

Using propensity score matching with two matches per HCV diagnosis, differentiating factors between patients with and without hepatitis C were revealed: A higher median age in the HCV-group and the relative frequency of HCV-infections within two digit zip code areas were significant differentiators. However, all socio-medical variables acted in concert to predict a potentially elevated HCV-risk.

## Discussion

Using an ANN analysis of a large set of socio-medical data provided by two German health insurance companies we were able to identify high dimensional similarities within a group of patients with chronic hepatitis C. All 2544 confirmed HCV-infections could be relocated in a population of more than 1.8 million insurants within a small cluster. Moreover, 60% of the patients newly diagnosed in 2014 showed distinct coordinates within the ANN map. This group is of interest since the treatment data of these patients were collected and documented prior to the HCV diagnosis excluding a bias of the characteristics describing the treatment courses.

An additional group of 2217 patients without a confirmed HCV-diagnosis showed great similarities with the cohort of known HCV-infections. This number of suspected HCV-infections was small and in the magnitude of the estimated number of unknown cases indicating a tight screening efficacy of the ANN.

The total number of confirmed and suspected HCV-infections in the population studied, reached 0.26%, lower than the estimated HCV antibody and viremic prevalence in Germany of up to 0.9% and 0.65% respectively. The overall low prevalence could be explained by a recruitment bias since the data were provided by two private health insurance companies covering a high proportion of public servants.

Although our ANN analysis showed a robust clustering of confirmed HCV diagnoses within a large population further validation of the prediction model by testing (or even offering testing to) patients at risk for hepatitis C was not possible due to restrictions imposed by the ethics committee. Therefore, propagating the predictive algorithm through public relations, application software (“Apps”) or webpage algorithms is currently the only way to rise awareness for screening patients at risk for hepatitis C. However, the complex interplay of several variables and parameters constituting a potentially heightened HCV risk makes the formulation of a screening directive challenging. While a higher median age and the relative frequency of HCV-infections within two digit zip code areas were significant differentiators, other variables such as diagnosis codes and prescription patterns collaborated to predict a potentially elevated HCV-risk. Some of these predictors may have a comprehensible basis, e.g. the median age of HCV-infected patients most likely reflects the highest infection rates before 1991 when HCV screening tests became available. This is in accordance with screening programs initiated in the USA [11, 12]. Other factors like diagnosis codes or drug prescriptions exert more subtle effects only in conjunction. Wolffram et al. used a conventional screening approach including a questionnaire covering 16 guideline adapted risk scenarios. The HCV-RNA prevalence in 21,008 patients was 0.43%; 65% of these infections were previously unknown. Since only established risk factors were evaluated, the most common scenarios such as IV drug use, blood transfusion before 1992 and immigration were significantly associated with hepatitis C [13].

A strength of this study is the large dataset containing socio-medical variables of more than 1.8 million insurers as well as the powerful data mining methodology using an ANN. However, our study has also limitations: First, we were not able to validate the prediction model by testing patients with a presumed risk of HCV-infection due to requirements given by the ethics committee. Second, the data were provided by two German private health insurances, thereby introducing a selection bias limiting the implementation for other national and international populations. Third, the learning variables used to train the ANN will most likely have to be modified according to the data available and population settings.

## Conclusions

In summary, we developed a prediction model for identifying patients with a potentially elevated risk of hepatitis C virus infection using an artificial network based data mining approach on a large dataset of socio-medical data. Further investigation is required to validate this new approach on screening for unidentified hepatitis C virus infections.

## Abbreviations

ANN: artificial neural network
ATC: anatomical therapeutic chemical classification system
DAA: direct-acting antivirals
DEBEKA: Debeka Krankenversicherungsverein a. G.
GHSS: Global Health Sector Strategy
HCV: hepatitis C virus
HUK Coburg: HUK-COBURG-Krankenversicherung AG
PZN: Pharmazentralnummer
SOM: self organizing map
